# Aldehyde dehydrogenase 1–positive nigrostriatal dopaminergic fibers exhibit distinct projection pattern and dopamine release dynamics at mouse dorsal striatum

**DOI:** 10.1038/s41598-017-05598-1

**Published:** 2017-07-13

**Authors:** Carmelo Sgobio, Junbing Wu, Wang Zheng, Xi Chen, Jing Pan, Armando G. Salinas, Margaret I. Davis, David M. Lovinger, Huaibin Cai

**Affiliations:** 1 Laboratory of Neurogenetics, Transgenic Section, National Institute on Aging, National Institutes of Health, Bethesda, MD 20892 USA; 2 Laboratory for Integrative Neuroscience, Section on Synaptic Pharmacology, National Institute on Alcohol Abuse and Alcoholism, National Institutes of Health, Rockville, MD 20852 USA

## Abstract

Aldehyde dehydrogenase 1 (ALDH1A1)–positive dopaminergic (DA) neurons at the ventral *substantia nigra pars compacta* (SNpc) preferentially degenerate in Parkinson’s disease (PD). Their projection pattern and dopamine release properties, however, remains uncharacterized. Here we show that ALDH1A1–positive axons project predominantly to the rostral two–thirds of dorsal striatum. A portion of these axons converge on a small fraction of striosome compartments restricted to the dorsolateral striatum (DLS), where less dopamine release was measured compared to the adjacent matrix enriched with the ALDH1A1–negative axons. Genetic ablation of *Aldh1a1* substantially increases the dopamine release in striosomes, but not in matrix. Additionally, the presence of PD-related human α-synuclein A53T mutant or dopamine transporter (DAT) blockers also differentially affects the dopamine output in striosomes and matrix. Together, these results demonstrate distinct dopamine release characteristics of ALDH1A1–positive DA fibers, supporting a regional specific function of ALDH1A1 in regulating dopamine availability/release in striatum.

## Introduction

ALDH1A1 belongs to a large family of aldehyde dehydrogenases that oxidize a variety of reactive aldehyde species^1^. ALDH1A1 is predominantly expressed by a subgroup of DA neurons in the midbrain^2^. In DA neurons, ALDH1A1 mediates the oxidation of the cytotoxic dopamine intermediate, 3, 4-dihydroxyphenylacetaldehyde (DOPAL), to the less reactive 3, 4-dihydroxyphenylacetic acid (DOPAC)^3, 4^. ALDH1A1 was also involved in the synthesis of retinoic acid (RA), which plays a crucial role in neuronal patterning, differentiation and survival^5^. Most recently, ALDH1A1 is reported to mediate the synthesis of the inhibitory neurotransmitter γ-aminobutyric acid (GABA) in DA neurons^6^. Intriguingly, two subpopulations of DA neurons at SNpc have been segregated along the dorsal to ventral axis for their ability to express ALDH1A1^7^. DA neurons located in the dorsomedial SNpc lack ALHD1A1 expression, whereas DA neurons in the ventrolateral tier express this enzyme. We recently demonstrated that ALDH1A1–positive DA neurons are more susceptible to degeneration in PD^8^. However, little is known about the connectivity and functionality of this group of neurons. It is unclear whether the axons of these ALDH1A1–expressing DA neurons terminate at distinct locations at the dorsal striatum and exhibit different dopamine release dynamics.

Previous neuron tracing studies suggest that DA neurons at the dorsal tier of SNpc project to the matrix compartment of dorsal striatum, whereas neurons at the ventral tier send their axons to the complementary striosome or patch compartment^9^. Striosome and matrix compartments are distinguished by expression of different biochemical markers, their embryonic development, and afferent and efferent connectivity^10, 11^. Striosomes are characterized by enhanced expression of µ opiate receptor (MOR1)^12^, enkephalin^13^, and dopamine transporter DAT^14^. In contrast, matrix is rich in acetylcholinesterase^12^ and calbindin^15^. Three different types of axon fibers have been described within the nigrostriatal projections^14^. Type A fibers arise from the dorsal tier of SNpc and target mainly to the matrix compartment of dorsal striatum. Type B fibers are from the ventral tier DA neurons and terminate predominantly in the striosomes. Considering ALDH1A1–positive DA neurons are mainly distributed at the ventral SNpc, it is reasonable to assume that they project mainly to the striosomes. However, the precise projection pattern remains to be determined. Dopamine release also appears to be different in the striosome and matrix compartments of dorsal striatum^16^. As we demonstrated recently, dopamine release at striosomes is lower compared to the matrix^16, 17^. However, more study is required to understand how the transmitter release is differentially regulated.

The goal of the present study aims to determine the projection pattern and dopamine release properties of ALDH1A1–positive DA axons at the dorsal striatum. Since overexpression of PD-related α-synuclein A53T missense mutation substantially impairs dopamine release at the striatum^18^, we additionally examined whether DA release at ALDH1A1–positive and –negative axon terminals are differentially affected by mutant α-synuclein.

## Results

### ALDH1A1–positive DA axon fibers project unevenly to the dorsal striatum

**Figure 1:**
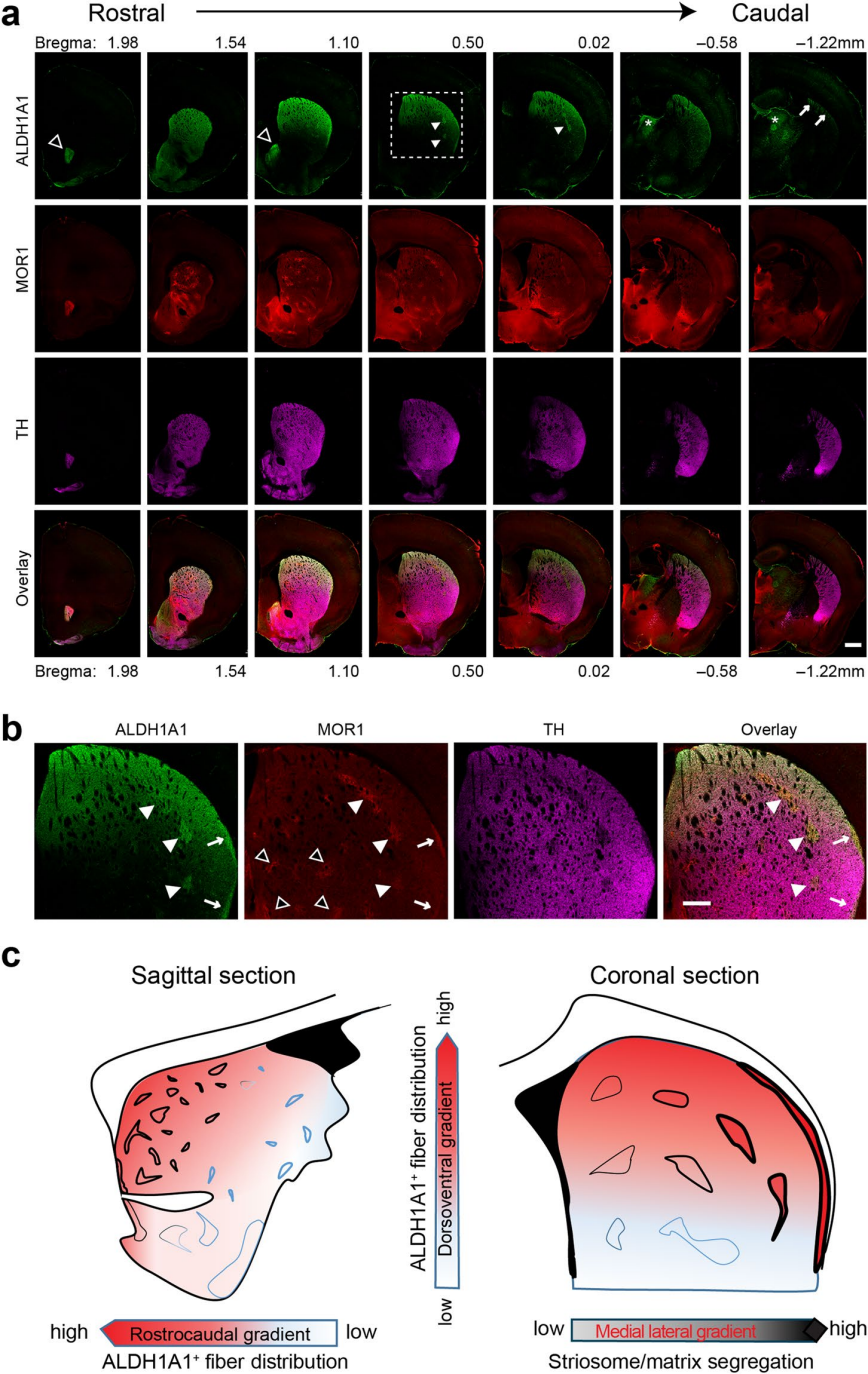
Distinct projection pattern of ALDH1A1–positive DA axons at striatum. (**a**) Representative images show ALDH1A1 (green), MOR1 (red), and TH (purple) co-staining in sequential striatal coronal sections of 1-month-old mice. Open arrowheads point to nucleus accumbens shell. Solid arrowheads indicate the striosomes. Arrows identify subcallosal streaks. Asterisks mark bundles positive to ALDH1A1 staining, but negative to TH staining. Scale bar: 500 µm. (**b**) Enlarged images of boxed area (**a**) show co-staining of ALDH1A1 and MOR1 in strisomes (solid arrowheads) and subcallosal streaks (arrows) at DLS, but not in striosomes (open arrow) at DMS. Scale bar: 100 µm. (**c**) Schematic diagram outlines the projection pattern of ALDH1A1–positive SNpc DA axon fibers in dorsal striatum.

Previous neuron tracing studies suggest that DA neurons at the ventral tier of SNpc send their axons to the striosome compartment of dorsal striatum^9^. However, there is a lack of molecular markers to reliably identify these neurons and their projections. Since ALDH1A1 is predominantly expressed by the ventral SNpc DA neurons in the brain^2, 8, 19, 20^, we stained sequential coronal sections of 1–month–old wild-type C67BL6 mice to examine the projection pattern of ALDH1A1–positive SNpc axon fibers at the striatum using antibodies against ALDH1A1, the striosomal marker MOR1, and the DA axon terminal marker tyrosine hydroxylase (TH). We found that ALDH1A1–positive DA fibers project unevenly along the rostrocaudal, dorsoventral, and mediolateral axis (Fig. 1a). In the rostral one–third (Bregma: ~2 to 1 mm), ALDH1A1–positive DA fibers mainly terminated at the medial part of nucleus accumbens shell (open arrowheads, top row), and the dorsal half of dorsal striatum. More condensed fibers were observed at the superficial layers of dorsal striatum, whereas no apparent segregation to the striosome or matrix compartments was found. In the middle one–third (Bregma: ~1 to 0 mm), ALDH1A1–positive DA fibers predominantly projected to the most dorsal layers of dorsal striatum. In the dorsomedial striatum (DMS), projections onto striosomes and matrix were not clearly segregated (Fig. 1a,b). By contrast, in the dorsolateral striatum (DLS), ALDH1A1–positive DA fibers largely converged to the most dorsal striosomes (arrowheads) and subcallosal streaks (arrows) (Fig. 1b). Finally, in the caudal one–third of striatum (Bregma: ~0 to –1.2 mm), there were fewer ALDH1A1–positive DA fibers projections, where they were present mainly in the superficial layers of DLS (Fig. 1a). Noticeably, there were a few clusters of ALDH1A1–positive fiber like structures in the thalamus, but they were negative for TH staining (arrows, top row, Fig. 1a). Furthermore, the projections only converge at the DLS striosomes and subcallosal streaks in the middle segment of dorsal striatum. The same projection pattern of ALDH1A1–positive DA fibers in the striatum was also observed in 3-month-old mice (Supplementary Fig. S1). To further specify the origin of ALDH1A1–positive signals in the striatum, we injected *Aldh1a1* siRNA plus green florescent protein (GFP)–expressing recombinant adeno-associated viruses^6^ in the SNpc in one hemisphere of 3-month-old wild type mice (Supplementary Fig. S2a). In line with the previous publication^6^, this AAV vector effectively inhibited ALDH1A1 expression in the SNpc (Supplementary Fig. S2a). More importantly, the ALDH1A1 staining was substantially suppressed in the ipsilateral dorsal striatum marked by GFP signals (Supplementary Fig. S2b). Taken together, these results demonstrate an uneven projection pattern of ALDH1A1–positive DA fibers in striatum, in which the innervation appears to follow a decreased gradient along the rostrocaudal and dorsoventral axis (Fig. 1c).

### ALDH1A1–positive fibers release less dopamine at the DLS striosomes

**Figure 2:**
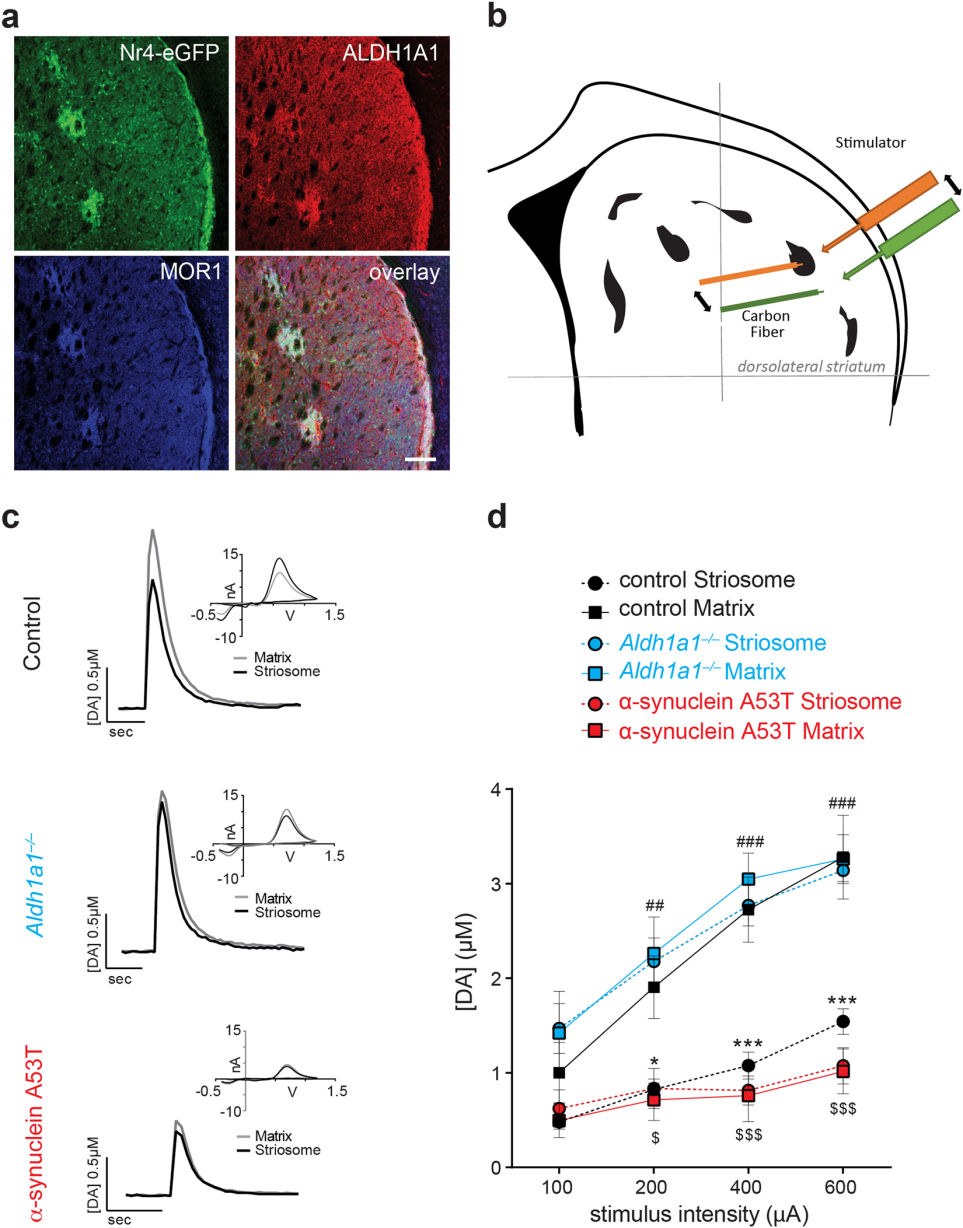
Different dopamine release properties of ALDH1A1–positive and negative fibers at DLS. (**a**) Representative images show GFP (green), ALDH1A1 (red), and MOR1 (purple) staining at DLS of Nr4a1-GFP mice on control *Aldh1a1* ^+/+^ background. Scale bar: 100 µm. (**b**) Schematic diagram of electrode placement in acute striatal slice for FSCV recording. Distances between electrodes (~100 µm) and from corpus callosum were maintained constant for dopamine release recording in striosome (orange) and matrix (green). (**c**) Representative traces of electrically evoked dopamine release in dorsolateral striatal compartments of control, *Aldh1a1* ^*−/*−^, and α-synuclein A53T mice, with current and voltage (CV) curve as inset. (**d**) Input/Output curves of peak dopamine release in dorsolateral striatal compartments of control, *Aldh1a1* ^*−/*−^, and α-synuclein A53T mice (n = 5 animals per genotype, 7 to 10 striosomes-matrix pairs per animal). Data were presented as mean ± SEM. Two-way ANOVA was used for statistical analysis, followed by Bonferroni’s *post hoc* tests.

We recently characterized a line of transgenic mice that express GFP mainly in the striatal neurons at striosomes under the transcriptional control of nuclear receptor Nr4a1^21^. Consistent with this early finding, the GFP signals overlapped with ALDH1A1 and MOR1 staining in the DLS striosomes of 3-month-old Nr4a1–GFP transgenic mice (Fig. 2a). Using GFP as a visible striosomal marker using fluorescence microscopy, we measured the dopamine release of ALDH1A1–positive fibers at the striosomes and ALDH1A1–negative fibers in adjacent matrix by fast scan cyclic voltammetry (FSCV) (Fig. 2b). In confirmation of our previous observations^16^, the magnitude of afferent stimulus-evoked DA release amplitudes was substantially lower in the DLS striosomes than the surrounding matrix areas in Nr4a1-GFP mice with the *Aldh1a1* wild-type (*Aldh1a1* ^+/+^) background (Fig. 2c,d; ANOVA interaction. Stim. Intensity × Compartment: F_(3,30)_ = 5.363, p < 0.01; Bonferroni’s post hoc test: ^*^p < 0.05, ^***^p < 0.001). Meanwhile, no obvious alteration of τ was found (for stimulation of 200 µA, Matrix: 0.4773 ± 0.08174 Vs Striosome: 0.4283 ± 0.06379; t_(10)_ = 0.4726 p > 0.05). These data suggestdifferent dopamine release in ALDH1A1–positive fibers that converge to the DLS striosomes.

### ALDH1A1 regulates dopamine release at the DLS striosomes

To evaluate the contribution of ALDH1A1 activity to DLS striosomal DA release, we performed FSCV in Nr4a1-GFP mice crossbred into the *Aldh1a1* knockout (*Aldh1a1* ^−/−^) background. As shown in the I/O curves, ALDH1A1-deficiency led to increases of DA release peak amplitude selectively in striosomes (Fig. 2c,d; ANOVA Interaction for Genotype × Stim. Intensity × Compartment: F_(15,112)_ = 1.925, p < 0.05; Post hoc test for striosomal area compared to controls: ^##^p < 0.01, ^###^p < 0.001). These results demonstrate that ALDH1A1 actively regulates dopamine release in ALDH1A1–positive fibers projecting to the DLS striosomes, but not the surrounding matrix area.

### Overexpression of human α–synuclein A53T mutation suppresses DA release mainly in matrix

We previously observed a drastic reduction of dopamine release at the dorsal striatum of α-synuclein A53T transgenic mice^18^. The transgenic α-synuclein was uniformly expressed throughout TH–positive fibers innervating striosomes and matrix (Supplementary Fig. S3). However, we do not know whether the presence of PD-related mutated α-synuclein affects the dopamine release similarly at the ALDH1A1–positive and negative terminals. Here we crossbred Nr4a1-GFP mice with the α-synuclein A53T transgenic mice to mark the ALDH1A1–positive terminals in the DLS striosomes. Data collected from the α-synuclein A53T mice revealed a substantial reduction of dopamine release in the matrix, while the striosomes were largely spared (Fig. 2c,d; ANOVA interaction Genotype × Stim. Intensity × Compartment: F_(9, 60)_ = 7.763, p < 0.001). These data indicate that ALDH1A1–positive DA fibers are more resistant to α-synuclein overexpressioninduced dopamine release impairments compared to the negative ones.

### DAT blockade differentially affects dopamine release at striatal compartments in *Aldh1a1*^+/+^, *Aldh1a1*^−/−^ and α-synuclein A53T mice

**Figure 3:**
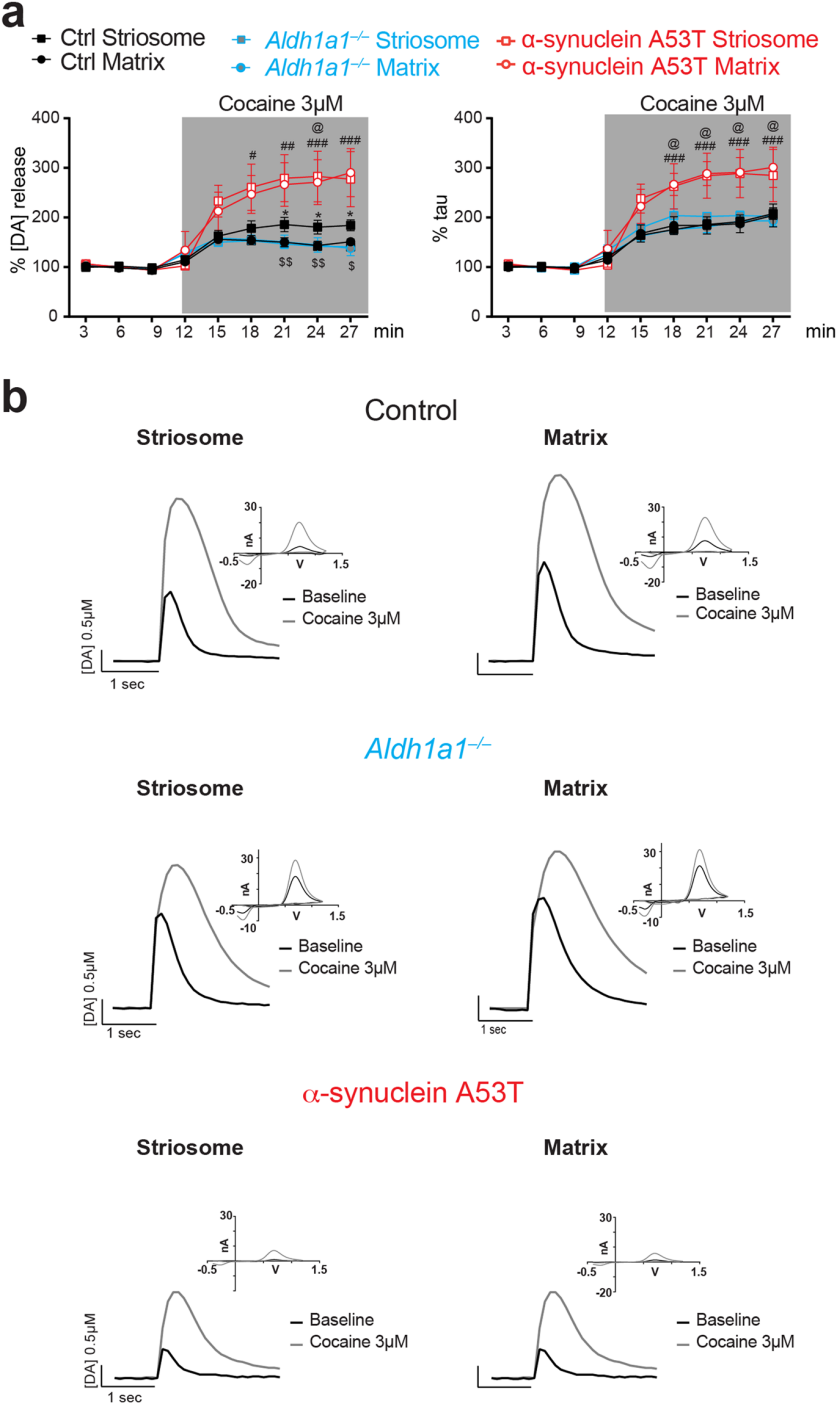
DAT blockade differentially affects dopamine release in ALDH1A1–positive and negative axons at DLS of control and mutant mice. (**a**) Percentages of electrical evoked dopamine amplitude (left) and uptake time constant (right) normalized to their baseline values (n = 5 animals per genotype, 2 sections per animal). Data were presented as mean ± SEM. Two-way ANOVA was used for statistical analysis, followed by Bonferroni’s *post hoc* tests. (**b**) Representative traces of electrically evoked dopamine release in DLS sub-compartments of control, *Aldh1a1* ^−/−^ and α-synuclein A53T mutant mice, with CV curve as inset.

Our previous study demonstrated that DAT blockade differentially potentiates peak DA release in striosome and matrix compartments^16^. We confirmed this finding in which the DAT blocker, cocaine, enhanced dopamine release more in the striosomes than in the proximal matrix areas in control mice (Fig. 3a,b; left panel: ANOVA interaction Stim. Intensity × Compartment: F_(8, 64)_ = 3.187, p < 0.01; Bonferroni’s post hoc Test ^*^p < 0.05). However, this inter-compartmental difference was not detectable in the *Aldh1a1* ^−/−^ mice (Fig. 3a,b; left panel: ANOVA interaction Genotype × Stim. Intensity × Compartment: F_(24, 128)_ = 2.880, p < 0.001; Bonferroni’s post hoc Test: striosomes ^$^p < 0.05, ^$$^p < 0.01). A similar lack of inter-compartmental difference was observed in α-synuclein A53T mutant mice, where both matrix and striosomes showed increased DA release to a similar extent after cocaine application (Fig. 3a,b; left panel: ANOVA interaction Genotype × Stim. Intensity × Compartment: F_(24, 128)_ = 2.880, p < 0.001; Bonferroni’s post hoc Test: matrix ^#^p < 0.05, ^##^p < 0.01, ^###^p < 0.001; striosomes ^@^p < 0.05). On the other hand, no inter-compartmental difference in τ was observed following DAT blockage in any of the three mouse lines (Fig. 3a,b; right panel). It is worth noting that after DAT blockade the τ was significantly higher in α-synuclein A53T mutant mice compared to *Aldh1a1* ^+/+^ mice (Fig. 3a, right panel; ANOVA interaction Genotype × Stim. Intensity × Compartment: F_(24, 128)_ = 2.603, p < 0.001; Bonferroni’s post hoc Test: matrix ^###^p < 0.001; striosomes ^@^p < 0.05). These results suggest different functional properties of DAT located at *Aldh1a1* ^+/+^ and *Aldh1a1* ^−/−^ axon terminals, suggesting that changes in ALDH1A1 and α–synuclein expression can alter DAT function in axon terminals.

### Dopamine D2 receptors modulate DA release similarly between compartments in *Aldh1a1*^+/+^, *Aldh1a1*^−/−^ and α-synuclein A53T mice

**Figure 4:**
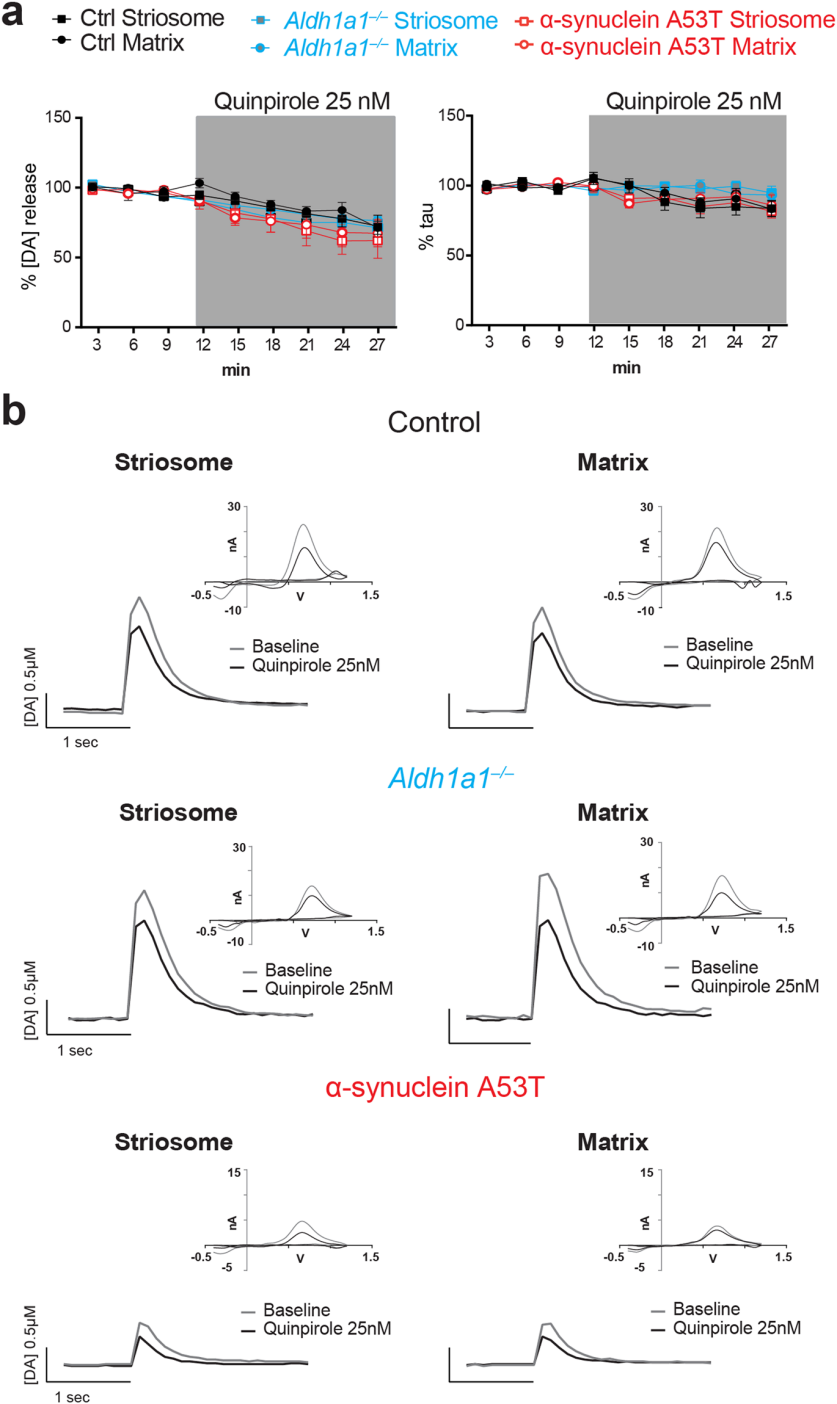
DRD2 autoreceptors modulate dopamine release similarly between ALDH1A1–positive and negative axons at DLS. (**a**) Percentages of electrical evoked dopamine amplitude (left) and uptake time constant (right) normalized to their baseline values (n = 5 animals per genotype, 2 sections per animal). Data were presented as mean ± SEM. Two-way ANOVA was used for statistical analysis, followed by Bonferroni’s *post hoc* tests. (**b**) Representative traces of electrically evoked dopamine release in DLS sub-compartments of control, *Aldh1a1* ^−/−^ and α-synuclein A53T mutant mice, with CV curve as inset.

D2 autoreceptors (DRD2s) provide a a presynaptic feedback system that regulates DA neurotransmission in striatum^22, 23^. We examined the ability of the DRD2 agonist quinpirole to depress dopamine release in slices from the different mouse lines. Application of quinpirole (25 nM) decreased dopamine release amplitude to a similar extent in *Aldh1a1* ^+/+^, *Aldh1a1* ^−/−^ and α-synuclein A53T mice, with little effect on τ in all groups (Fig. 4a,b). These data suggest the presence of ALDH1A1 or mutant α-synuclein do not affect the function of presynaptic DRD2s that alter DA release.

### Nicotinic acetylcholine receptors (nAChRs) have no regionally specific effect on dopamine dynamics in striosomes and matrix, but have reduced effects on striatal dopamine release in *Aldh1a*^−/−^ and α-synuclein A53T mice

**Figure 5:**
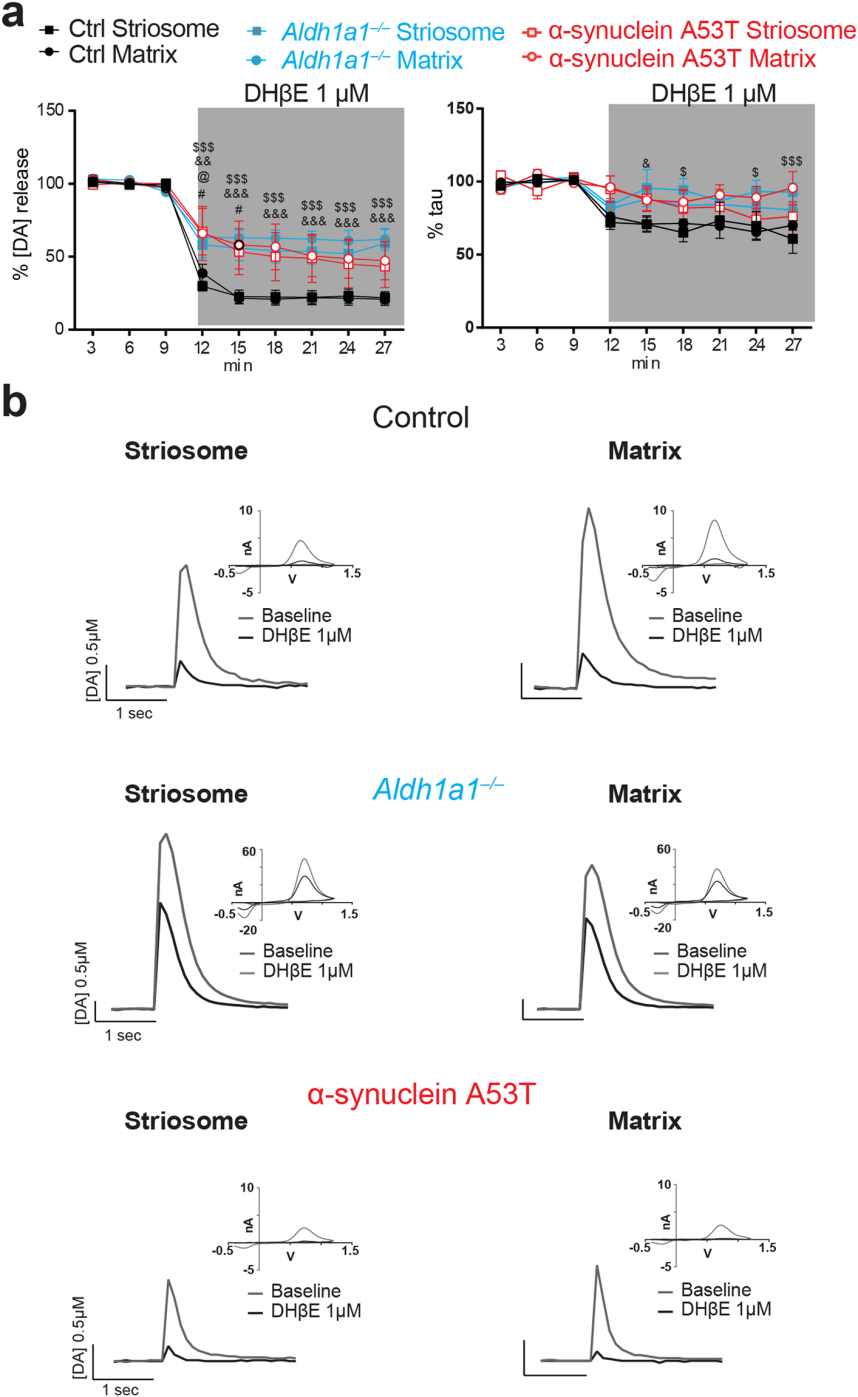
Nicotinic acetylcholine blockade does not have differential effects on DA release from ALDH1A1–positive and negative axons, but is less effective in both *Aldh1a1* ^−/−^ and α-synuclein A53T mutant mice. (**a**) Percentages of electrical evoked dopamine amplitude (left) and uptake time constant (right) normalized to their baseline values (n = 5 animals per genotype, 2 sections per animal). Data were presented as mean ± SEM. Two-way ANOVA was used for statistical analysis, followed by Bonferroni’s *post hoc* tests. (**b**) Representative traces of electrically evoked dopamine release in DLS sub-compartments of control, *Aldh1a1* ^−/−^ and α-synuclein A53T mutant mice, with CV curve as inset.

Striatal nAChRs influence many aspects of striatal function and related behavior, in part through a strong interaction with the DA system^24^. ACh stimulates dopamine release through nACh receptors (nAChRs) on DA axons, mainly via β2-subunit-containing receptors^25, 26^. We found that DHβE (1 μM), an antagonist ofpresynaptic β2-subunit-containing nAChRs on DA terminals, had the same inhibitory effect on dopamine release in both striatal compartments, but produced a smaller magnitude effect in *Aldh1a1* ^−/−^- and A53T mutants (Fig. 5a, left panel; Fig. 5b). Both compartments of *Aldh1a1* ^−/−^ mice showed lower DHβE inhibition compared with control littermates (ANOVA interaction Compartment × Time: F_(24, 128)_ = 8.71; Bonferroni’s post hoc test: Striosomes: ^$$$^p < 0.001; Matrix: ^&&^p < 0.01, ^&&&^p < 0.001). In A53T mutant mice, the effect of presynaptic nAChR blockade was also proportionally lower than in control (Fig. 5a, left panel; ANOVA interaction Time × Compartment: F_(24, 128)_ = 2.061, p < 0.01; Bonferroni’s post hoc test: Control vs α-synuclein A53T: Matrix: ^#^p < 0.05; Striosomes: ^@^p < 0.05). Meanwhile, the DHβE-induced reduction in τ was significantly reduced in controls in both *Aldh1a1* ^−/−^ (Fig. 5a, right panel; ANOVA interaction Time × Compartment: F (24, 128) = 2.301, p < 0.01; Bonferroni’s post hoc test: Striosomes ^$^p < 0.05, ^$$^p < 0.01, ^$$$^p < 0.001; Matrix ^&^p < 0.05) and A53T mutant mice (Fig. 5a, right panel; ANOVA interaction Time × Compartment: F (24, 128) = 1.636, p < 0.05. Bonferroni’s post hoc test: Matrix: ^#^p < 0.05). These results suggest that dopamine release in the DLS compartments of *Aldh1a1* ^−/−^- and A53T mice is less affected by the inhibition of β2-nAChR.

### ALDH1A1–positive DA axon fibers show differential expression of TH, DAT, and VMAT2 in striosomes and matrix

**Figure 6:**
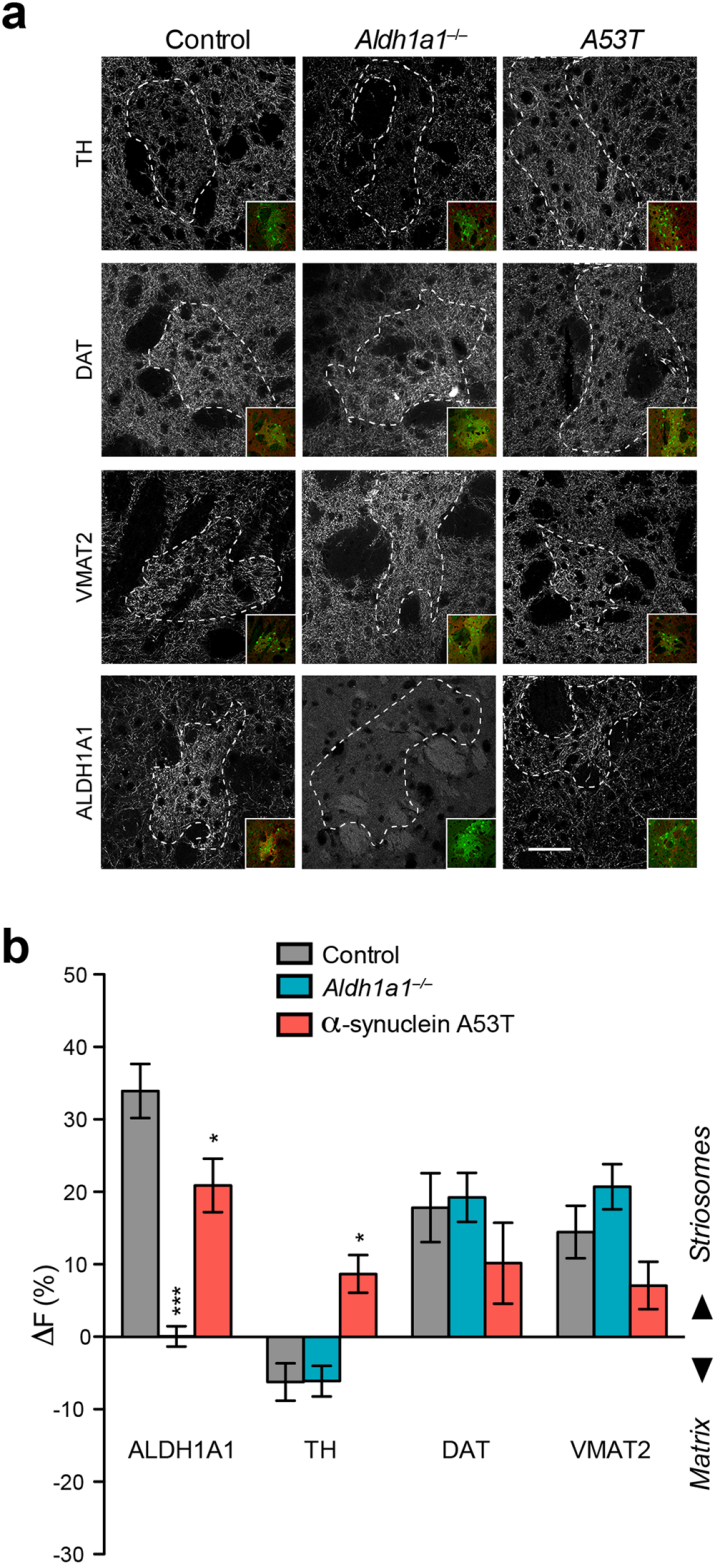
Distribution of TH, DAT, VMAT2, and ALDH1A1 proteins in the striosomes and matrix of DLS. (**a**) Representative images show TH, DAT, VMAT2, and ALDH1A1 staining in the DLS of 1-month-old Nr4a-GFP mice in control, *Aldh1a1* ^−/−^ and α-synuclein A53T mutant background. Dashed lines outline the striosomes. Inserts show co-staining with GFP to mark the strisomes. Scale bar: 50 µm. (**b**) Bar graph depicts the relative fluorescence intensity (ΔF) of ALDH1A1, TH, DAT, and VMAT2 staining in the striosome and matrix compartments (n = 5 animals per genotype, x matrix-striosome pairs per animal). Data were presented as mean ± SEM. One-way ANOVA was used for statistical analysis for each protein, followed by Bonferroni’s *post hoc* tests where *Aldh1a1* ^−/−^ and α-synuclein A53T mutant were compared to Controls (*p < 0.05, ***p < 0.001).

Given that TH, DAT, vesicular monoamine transporter 2 (VMAT2), and ALDH1A1 are key proteins participating in synthesis, transport, and catabolism of dopamine^27–29^, we examined their expression to explore the underlying molecular mechanisms of differential dopamine release dynamics of ALDH1A1–positive fibers in the DLS. Using the same NR4a1-GFP reporter to outline the striosomes, we co-stained the middle one–third of striatal coronal sections of 1–month–old *Aldh1a1* ^+/+^, *Aldh1a1* ^−/−^, and α-synuclein A53T mice with antibodies against ALDH1A1, TH, DAT, and VMAT2 (Fig. 6a). The intensities of ALDH1A1, DAT, and VMAT2–positive fluorescence signals were substantially higher in the striosomes compared to the adjacent matrix compartments (Fig. 6a,b). A similar weaker TH staining was also observed in the DLS striosomes of 3-month-old mice (Supplementary Fig. S1). By contrast, the intensity of TH staining was weaker in the striosomes (Fig. 6a,b). The absence of *Aldh1a1* did not alter the overall distribution of TH, DAT and VMAT2 between striosomes and matrix (Fig. 6a,b). On the other hand, expression of the transgenic α-synuclein A53T mutant protein increased the expression of TH but suppressed the expression of ALDH1A1 (ANOVA Genotype × Protein Interaction: F (3, 73) = 5.576; p < 0.01; Bonferroni’s post hoc test: ^*^p < 0.05) but not significantly for DAT and VMAT2 (Fig. 6a,b). The differential expression of ALDH1A1, TH, at the ALDH1A1–positive fibers may contribute to the distinct dopamine release dynamics in the DLS striosomes.

## Discussion

It has been well documented that DA neurons located at the ventrolateral tier of SNpc preferentially degenerate in the postmortem brains of PD patients^30, 31^. Recently, we demonstrated that those DA neurons selectively express ALDH1A1, a multi-functional aldehyde dehydrogenase^8^. Given that ALDH1A1 is predominantly expressed by the midbrain DA neurons^2, 8, 19, 20^, the appearance of ALDH1A1 staining in the dorsal striatum most likely reflects the trafficking of ALDH1A1 proteins to the axon terminals. Indeed, ALDH1A1 signals were co-localized with the DA neuron marker TH in the striatum, but not with the striosomal neuron marker MOR1. ALDH1A1 staining reveals a more complex projection pattern of ventral SNpc DA neurons compared to early neuron tracing studies^9^. While the cell bodies of ALDH1A1–positive DA neurons mostly reside at the ventral tier of SNpc, the axon fibers more heavily innervate the most dorsal portions of dorsal striatum. Furthermore, ALDH1A1–positive DA axon fibers only show a clear convergence in the striosomes in the DLS. The more homogenous projections to DMS likely result from the presence of overlapping DA innervation from ALDH1A1–positive DA neurons in the VTA^20^, likely the same DA neurons involved in a precise form of reward prediction^32^. DLS-projecting SNpc DA neurons have been implicated in motor control and show similar responses to both rewarding and aversive stimuli^33^. DMS-projecting neurons, on the other hand, respond differently to rewarding and aversive inputs^33, 34^. Future studies will need to further distinguish subpopulations of ALDH1A1–positive SNpc DA neurons based on their distinct molecular composition, connectivity and functionality.

Degeneration of DLS–projecting ALDH1A1–positive SNpc neurons is strongly implicated in the pathogenesis of PD^2, 30, 31, 35^. As an initial step to characterize the functional properties of these neurons, we systematically measured their dopamine release and reuptake parameters under different genetic and pharmacological manipulations. Normally, dopamine release amplitudes are lower in striosomes than matrix areas in control mice^16, 17^, which is correlated with a lower level of TH expression in the axons terminating in the striosomes of human^36^ and mouse brains, These observations uggest that TH expression level is a factor contributing to reduced dopamine release in DLS–projecting ALDH1A1–positive SNpc neurons. However, genetic ablation of *Aldh1a1* substantially increased DA release selectively in the DLS striosomes to a level that matches dopamine release to the adjacent matrix, arguing that ALDH1A1 plays a more important role in determining the inter-compartmental differences in dopamine release. This finding is also consistent with an early report that shows a higher than normal extracellular striatal dopamine level in *Aldh1a1* ^−/−^ mice as measured by *in vivo* microdialysis^27^. Since ALDH1A1 inhibition does not affect the overall expression of TH, DAT and VMAT2 proteins^27^ or their relative distributions in DA fibers ending in striosomes and matrix, ALDH1A1 likely limits the dopamine release through increasing metabolism of cytosolic dopamine^37^. Therefore, a lack of ALDH1A1 may promote cytosolic dopamine buildup, resulting in more vesicular loading and release upon stimulation. Noticeably, the expression of ALDH1A1 is substantially suppressed in α-synuclein A53T transgenic mice and PD brains^8^. The reduced ALDH1A1 expression, together with a relative increase of TH expression, likely serves as a compensatory mechanism to enhance dopamine release. Paradoxically, the loss of ALDH1A1 also leads to higher levels of highly reactive DOPAL intermediates, which are detrimental to the survival of DA neurons^2, 8, 38^. Understanding how ALDH1A1–positive fibers react to PD-related genetic and environmental insults could be informative to develop new therapeutic approaches in the treatment of PD^27^.

Increasing evidence suggests that α-synuclein mutations directly interfere with the synaptic vesicle release machinery and inhibit neurotransmission^39–41^. Compared to the other α-synuclein mouse models, this line of α-synuclein transgenic mice develop much more robust pathological abnormalities^18^. At one month of age, these α-synuclein transgenic mice show >15% loss of SNc DA neurons, and >80% reduction of dopamine release^18^. More α-synuclein aggregates were accumulated in the ALDH1A1-negative DA neurons compared to the positive ones^8^, which may contribute to the preferential loss of ALDH1A1-negative DA neurons in these mice. Although transgenic α-synuclein is expressed comparably by both ALDH1A1–positive and negative SNpc DA neurons^8^ and fibers, the reduction of dopamine release was more severe in the ALDH1A1-negative axons terminating in the matrix. The preferential degeneration of ALDH1A1–negative SNpc DA neurons and axon fibers^8^ likely contributes to the overall reduction of dopamine release in the matrix. The presence of excessive α-synuclein also substantially increased the effect of cocaine on the DA peak amplitude. The greater relative efficacy of cocaine to affect dopamine dynamics in the α–synuclein transgenic mice may be attributable to a compensatory increase in basal DAT activity following DA terminal loss in these mice^18^. In addition, under our experimental conditions we did not observe any difference in DRD2 autoreceptor-mediated inhibition of dopamine release in either striatal compartment by the DRD2. Therefore, the observed DAT adaptation may be independent of any indirect effect from DRD2 autoreceptor activity.

Striatal ACh influences many aspects of striatal function, and related behaviors, through a strong interaction with the DA system^24^. ACh stimulates DA release through nAChRs on DA axons, mainly via β2-subunit-containing receptors^25, 26^. As mentioned previously^16^, we did not observe a robust difference of presynaptic nAChR blockade between the DA terminals ended in striosomes and matrix, suggesting that this cholinergic effect has no preferential effects within a specific striatal compartment under current experimental conditions. However, dopamine release in the DLS of *Aldh1a1* ^−/−^ and α-synuclein A53T mutant mice was less affected by antagonism of β2-nAChRs. A floor effect may prohibit our ability to determine the efficacy of nAChR inhibition on DA release in α-synuclein transgenic mice, as well as any intercompartmental contrast. On the other hand, augmented extracellular dopamine in *Aldh1a1* ^−/−^ striata very likely hampers the ability of the local ACh system to enhance DA release, although more detailed investigation is needed to understand why *Aldh1a1* ^−/−^ DA neurons are less responsive to nAChR blockade.

In summary, our findings reveal a distinct projection pattern of an ALDH1A1–positive subpopulation of DA neurons to the dorsal striatum. We further characterized the dopamine release properties of a fraction of these axons that converge on the DLS striosomes, supporting an important role of ALDH1A1 in regulating dopamine availability and release.

## Methods

### Animal subjects

*Pitx3* ^+/IRES2–tTA^ knock–in mice and tetO–A53T α-synuclein transgenic mice were created as described previously^18, 42^. *Aldh1a* knockout (*Aldh1a1* ^−/−^) mice^43^ were obtained from the Jackson Laboratory (Bar Harbor, ME), and backcrossed with C57BL6J mice for more than five generations. Nr4a1–EGFP139Gsat (Nr4a1–GFP) transgenic mice^21^ were obtained from GENSAT and backcrossed with C57BL6J mice for more than five generations. The *Pitx3* ^+/IRES2–tTA^ mice were bred with Nr4a1–GFP mice for the generation of Nr4a1–GFP::*Pitx3* ^+/IRES2–tTA^ bigenic mice (referred as control mice), which then bred with tetO–A53T transgenic mice to generate Nr4a1–GFP::*Pitx3* ^+/IRES2–tTA^::tetO–A53T triple-transgenic mice (referred as α-synuclein A53T mice). Nr4a1–GFP mice were also crossbred with *Aldh1a1* ^−/−^ mice to generate Nr4a1–GFP::*Aldh1a1* ^*−/*−^ bigenic mice (referred as *Aldh1a1* ^−/−^ mice). All mice were housed in a 12-hour-light/dark cycle and fed regular diet ad libitum. The Animal Care and Use Committee (ACUC) of the National Institute on Aging, NIH approves all the mouse-related experimental procedures performed in this study. We carried out all the mouse experiments following the approved guidelines.

### Genotyping

Genomic DNA was prepared from tail biopsy using DirectPCR Lysis Reagent (Viagen Biotech Inc.) and subjected to PCR amplification using specific sets of PCR primers for each genotype, including *Pitx3* ^+/IRES2–tTA^ knock–in mice (Pitx3-F: GACTGGCTTGCCCTCGTCCCA and Pitx3-R: GTGCACCGAGGCCCCAGATCA), tetO–A53T transgenic mice (PrpEx2-F: TACTGCTCCATTTTGCGTGA and SNCA-R: TCCAGAATTCCTTCCTGTGG), *Aldh1a1* ^*−/*−^ mice (ALDH1A1muF: CTATCGCCTTCTTGACGAGTTCTT and ALDH1A1muR: CCTTGTACATCTTAACGGTGCACA), *Aldh1a1* wild-type (*Aldh1a1* ^+/+^) mice (ALDH1A1wtF: TAAAGACCTGGATAAGGCCATCA and ALDH1A1wtR: ACGGTGCACAAAATAAACATCTG), and Nr4a1–GFP mice were genotyped at birth by fluorescence of the thymus, tails and ears using a hand held LED light source.

### Stereotaxic viral injection

Stereotaxic AAV injections were conducted on 3–month–old C57BL6/J mice. Before surgery, mice were deeply anesthetized by intraperitoneal injection of ketamine (100 mg/kg)/xylazine (10 mg/kg) solution. A total volume of 500 nl virus solution was injected unilaterally into *Substantia Nigra pars compacta* (SNpc, coordinates used, AP: −1.5 mm, ML: ±0.9 mm from bregma, DV: −4.1 mm from exposed dura mater). Virus solution was injected at an infusion rate of 100 nl/min and withdrawn 10 min after the end of injection. Following virus injection, the scalp was sutured and mice were returned to their home cages. Virus-injected mice were used for experiment at least 4 weeks after virus infusion.

### Immunohistochemistry and light microscopy

Mice were anesthetized with ketamine and then transcardially perfused with a 4% formaldehyde/PBS solution as described previously^44^. The brains were then extracted and post fixed in 4% formaldehyde overnight, then submerged in 30% sucrose for 24 hours at 4 °C for later sectioning. Series of 40 μm sections were collected using a cryostat (Leica Biosystems, Richmond IL). The sections were blocked in a 10% normal donkey serum, 1% bovine serum albumin, 0.3% Triton X-100, PBS solution for 1 hour at room temperature on an orbital shaker at low speed. The sections were then incubated in a primary antibody/PBS solution over two nights as follows: goat anti-ALDH1A1 (1:1,000; R&D system), or rabbit anti-ALDH1A1 (1:1,000; Sigma-Aldrich), or rabbit anti-MOR1 (1:3,000; Immunostar), or mouse anti-TH (1:1,000; Immunostar), or rat anti-DAT (1:1,000; Millipore), or rabbit anti-VMAT2 antibody (1:50,000, obtained from Dr. Gary W. Miller) or mouse anti-human α-synuclein antibody (1:1,000; sc211, Santa Cruz). Sections were then washed three times in PBS before being incubated in secondary antibody solutions with Alexa 488- or Alexa Fluor 546–, or Alexa Fluor 633-conjugated secondary antibody (1:500, Invitrogen) at room temperature for 1 hour. Following three washes in PBS, sections were mounted onto subbed slides, and coverslipped with mounting media (ProLong® Gold Antifade Mountant, Life technology). Stained sections were imaged using a laser scanning confocal microscope (LSM 780; Zeiss). The paired images in the figures were collected at the same gain and offset settings. After collection, processing was applied uniformly to all paired images. The images were either presented as a single optic layer from individual fields or displayed as maximum intensity projections to represent confocal stacks.

### Image analysis

For the quantitative assessment of various marker proteins in striatum, the dorsal lateral striatum (DLS) patches were chosen randomly imaged by a 40× oil immersion lens from three mice per group. A single optical layer was taken using identical settings and exported to ImageJ (NIH) for imaging analyses. Images were converted to an 8-bit color scale (fluorescence intensity from 0 to 255) using ImageJ. Areas of interest, striosomes and adjacent matrix, were first selected by Polygon or Freehand selection tools and then subjected to measurement by mean optical intensities. The mean intensity for the background area was subtracted from the selected area to determine the net mean intensity.

### Fast-Scan Cyclic Voltammetry

FSCV was used to investigate dopamine release evoked by electrical stimulation in matrix and striosomal compartments localized in the dorsolateral quadrant of the striatum. Five mice of 10–14 weeks old from each of the three experimental groups (control, *Aldh1a1* ^−/−^ and A53T) were considered in this analysis. Since no significant difference was observed between Nr4a1–GFP and Nr4a1–GFP::*Pitx3* ^+/IRES2–tTA^ mice, the latter were considered as a valid control for all group comparisons. Coronal brain slices (250 μm) containing dorsal stratum from mice were prepared as described previously^45^. Slices were kept in oxygenated modified Kreb’s buffer as follows (in mM): NaCl 126, KCl 2.5, NaH2PO4 1.2, CaCl_2_ 2.4, MgCl_2_ 1.2, NaHCO_3_ 25, glucose 11, HEPES 20, L-ascorbic acid 0.4; at room temperature until required. Recordings were made at 32 °C in a chamber perfused at a rate of 1.5 ml/min. Cylindrical carbon-fiber microelectrodes (75–125 μm exposed fiber) were prepared with T650 fibers (6 μm diameter, Goodfellow) and inserted into a glass pipette. The carbon-fiber electrode was held at −0.4 V, and the potential was ramped to +1.2 V and back at 400 V/s every 100 ms. Dopamine (DA) release was evoked by a rectangular, electrical pulse stimulation (if not specified otherwise: 200 μA, 1.2 ms, monophasic) generated by a DS3 Constant Current Stimulator (Digitimer, Hertfordshire, UK). Delivery of an electrical stimulation was applied every 3 min by a concentrical bipolar electrode placed 100 μm from the recording electrode and always from matrix (Nr4a1–GFP–negative) area. Data collection was done using DEMON software^46^. Ten cyclic voltammograms of charging currents were recorded as background before stimulation, and the average was subtracted from data collected during and after stimulation. Matrix/striosome ratio of DA peak amplitude responses were obtained from six to ten striosome-matrix paired recordings (pseudo-randomly performed from individual striosomes and proximal matrix area) across the rostral-caudal extent of the dorsolateral striatum, and averaged for each single mouse. Input/output function (I/O) curves were constructed by plotting peak amplitude of DA release as a function of stimulus currentover a range of stimulus intensities. The time constant (τ) of the evoked DA response was used as an index of DA uptake. Two of the most representative striosome/matrix area pairs were chosen for each mouse, and selected randomly for pharmacological manipulation experiments.

### Drugs

Quinpirole, sulpiride, dihydro-β-erythrodine (DhβE) and cocaine hydrochloride were purchased from Sigma-Aldrich (St. Louis, MO, USA).

### Statistics

GraphPad Prism 6 (GraphPad Software, La Jolla, CA) was used for all statistical tests. Unpaired t-tests and two-ways ANOVA were used for the experiments examining dopamine release between groups and/or compartments, followed by Bonferroni’s post hoc test in case of significant interaction between factors, and reported in the figures.

## Electronic supplementary material

Supplementary Figures
