# Supplementary material for: Aldehyde dehydrogenase 1–positive nigrostriatal dopaminergic fibers exhibit distinct projection pattern and dopamine release dynamics at mouse dorsal striatum
Source: Sci Rep. 2017 Jul 13;7:5283. doi: 10.1038/s41598-017-05598-1 (PMC5509666; doi:10.1038/s41598-017-05598-1)
Supplement: Supplementary file 1 — Supplementary Figures [file 41598_2017_5598_MOESM1_ESM.pdf]

Aldehyde dehydrogenase 1–positive nigrostriatal dopaminergic fibers exhibit distinct projection pattern and dopamine release dynamics at mouse dorsal striatum

Carmelo Sgobio[1, #], Junbing Wu[1, #], Wang Zheng[1, #], Xi Chen[1], Jing Pan[1], Armando G. Salinas[2], Margaret I. Davis[2], David M. Lovinger[2], Huaibin Cai[1, *]

[1] Laboratory of Neurogenetics, Transgenic Section, National Institute on Aging, National Institutes of Health, Bethesda, MD 20892

[2] Laboratory for Integrative Neuroscience, Section on Synaptic Pharmacology, National Institute on Alcohol Abuse and Alcoholism, National Institutes of Health, Rockville, MD 20852

Supplementary Figures

Supplementary Figure S1

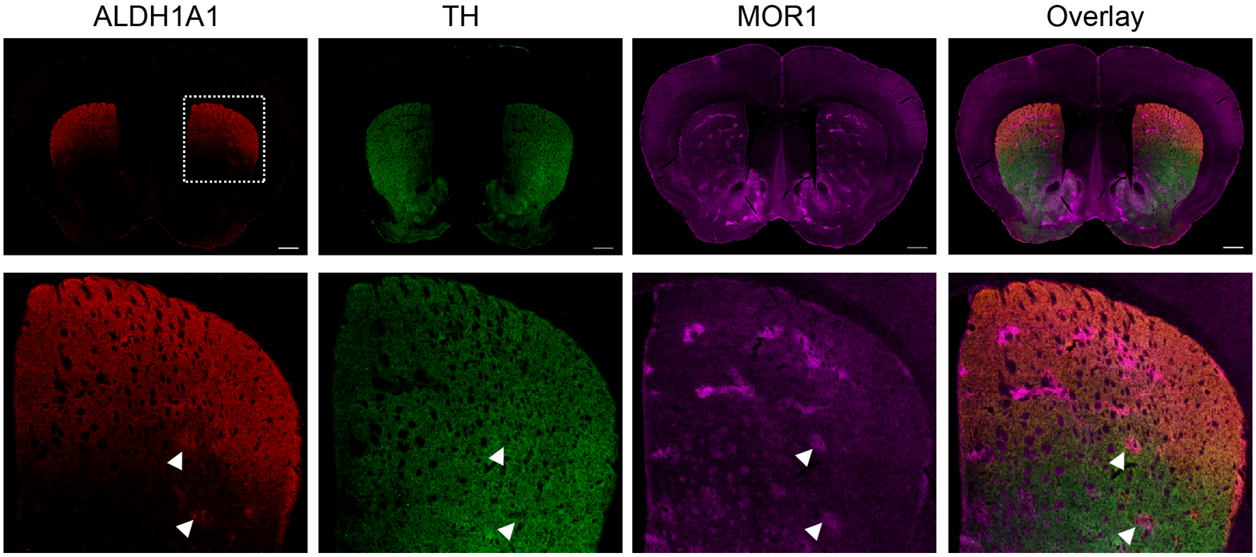

Fig. S1 Projection of ALDH1A1–positive DA fibers in the striatum of 3-month-old mice. Representative images show ALDH1A1 (red), TH (green), and MOR1 (purple) co-staining in striatal coronal sections of 3-month-old wild-type mice. Scale bar: 500µm. The bottom row highlights the boxed area in the top row. Solid arrowheads point to the ALDH1A1–positive fibers that converge to the DLS striosomes.

Supplementary Figure S2

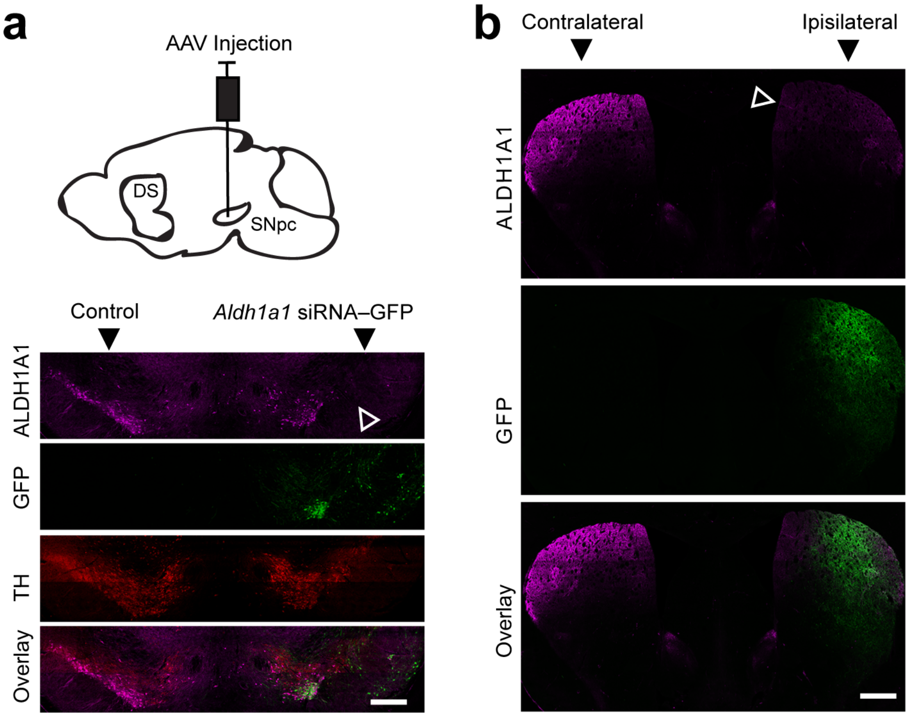

Fig. S2 ALDH1A1 staining at the dorsal striatum is derived from the ALDH1A1-expressing neurons in the SNpc. (a) The cartoon illustrates the stereotaxic injection of AAV into the SNpc in one side of hemisphere of 3–month–old wild type mice. Representative images show ALDH1A1 (purple), TH (red), and GFP (green) co-staining in a coronal section of SNpc. Open arrowhead points to the reduction of ALDH1A1 staining in the SNpc injected with *Aldh1a1* siRNA. Scale bar: 500µm. (b) Representative images display ALDH1A1 (purple), TH (blue), and GFP (green) co-staining in a striatal coronal section. The open arrowhead marks the substantial reduction of ALDH1A1 staining in the ipsilateral dorsal striatum (DS) indicated by the GFP signals. By contrast, the ALDH1A1 signals remains in the contralateral striatum. Scale bar: 500µm.

Supplementary Figure S3

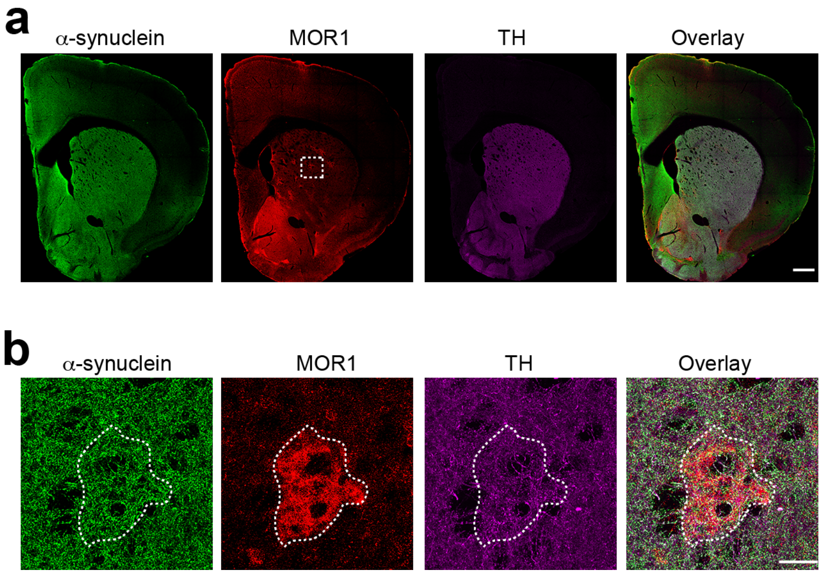

Fig. S3 Transgenic α-synuclein was uniformly expressed throughout TH-positive fibers innervating striosomes and matrix. (a) Representative images show human α-synuclein (green), MOR1 (red), and TH (purple) co-staining in striatal coronal sections of 4-month-old A53T mice. Scale bar: 500μm. (b) High magnification images of the boxed area in (a). The dash line outlines a striosome. Scale bar: 50μm.
